# Hormone-Specific Reprogramming of the Phenylpropanoid Network in Juvenile *Quercus sideroxyla* Leaves Revealed by Targeted Metabolomics

**DOI:** 10.3390/plants15040548

**Published:** 2026-02-10

**Authors:** Jessica Barragán-Zúñiga, Nuria Elizabeth Rocha-Guzmán, Jesús Omar Díaz-Rivas, Rubén Francisco González-Laredo, José Ángel Sigala-Rodríguez, José Alberto Gallegos-Infante, Martha Rocío Moreno-Jiménez

**Affiliations:** 1 Laboratorio Nacional CONAHCYT de Apoyo a la Evaluación de Productos Bióticos (LaNAEPBi), Unidad de Servicio Tecnológico Nacional de México, TecNM-I.T. de Durango, Blvd. Felipe Pescador 1830 Ote., Durango 34080, Mexico; barraganzj@gmail.com (J.B.-Z.); rubenfgl@itdurango.edu.mx (R.F.G.-L.); agallegos@itdurango.edu.mx (J.A.G.-I.); mrmoreno@itdurango.edu.mx (M.R.M.-J.); 2 Instituto de Investigación y Transferencia de Tecnología de Zonas Áridas, UAAAN, Parras de la Fuente 27980, Mexico; jesus.diaz@uaaan.edu.mx; 3 Instituto Nacional de Investigaciones Forestales, Agrícolas y Pecuarias, Campo Experimental Valle del Guadiana, Carretera Durango-El Mezquital, km 4.5, Durango 34170, Mexico; sigala.jose@inifap.gob.mx

**Keywords:** phenylpropanoid-metabolism, hormonal elicitation, *Quercus sideroxyla*

## Abstract

Phytohormones are key regulators of specialized metabolism, yet hormone-specific and time-dependent phenolic reprogramming in woody species remains poorly resolved. This study evaluated the phenolic responses of juvenile *Quercus sideroxyla* leaves grown under controlled greenhouse conditions to salicylic acid (SA), jasmonic acid (JA), and abscisic acid (ABA) using a pulse-based elicitation design combined with targeted metabolite profiling. Aqueous acetone extracts exhibited high phenolic diversity, including phenolic acids (20 compounds), flavonoids (15 compounds), and hydrolyzable tannins (27 compounds). Partial least squares–discriminant analysis (PLS-DA), multivariate statistics (MANOVA), and Random Forest classification were employed to resolve hormone-specific phenolic signatures across time. Distinct regulatory trajectories were identified for each hormone. SA elicitation triggered a high-amplitude, pulsatile phenolic response primarily affecting precursor-associated phenolic acids and ellagic-related structures, consistent with transient metabolic priming. JA induced a gradual and sustained modulation of flavonoids and tannin pools, within which advanced caffeoylquinic esters, flavonol conjugates, and ellagitannins emerged as key discriminant markers. ABA treatment promoted progressive and stabilized accumulation across phenolic classes, supporting a role in metabolic homeostasis rather than stress induction. Biweekly sampling was essential to discriminate transient, adaptive, and stabilizing responses. Overall, the results demonstrate that *Q. sideroxyla* differentiates hormonal signals and translates them into distinct phenolic reprogramming patterns. The integration of time-resolved metabolomics with multivariate and machine-learning approaches provides a robust framework for hormone-guided modulation of phenolic metabolism in woody plants.

## 1. Introduction

Phenolic compounds are a highly diverse group of plant secondary metabolites that play a vital role in various functions within the plant kingdom. They are critical to plant physiology, functioning as antioxidants, structural components, signaling molecules, and defense compounds against biotic and abiotic stress factors [1]. Phenolic acids and tannins are primarily derived from the phenylpropanoid pathway, whereas flavonoids originate from a specialized branch of this pathway through the flavonoid biosynthetic route. Together, these interconnected pathways generate a wide range of phenolic intermediates that modulate redox balance and plant adaptation to environmental stimuli [2]. Due to their ecological, physiological, and nutraceutical importance, phenolic profiles have become valuable indicators of biochemical responses and metabolic reprogramming in plants subjected to stress or elicitation.

*Quercus sideroxyla* Bonpl., commonly known as red oak, is a very abundant species in northern Mexico. It is ecologically significant in mountain ecosystems and shows promise as a source of bioactive phytochemicals. Extracts derived from its leaves are particularly rich in hydroxybenzoic and hydroxycinnamic acids, flavan-3-ols, and hydrolyzable tannins [3]. Within this group, hydrolyzable tannins, especially those related to ellagitannins, are characteristic of the *Quercus* genus and represent a chemically distinctive and functionally relevant fraction of the phenolic profile of the leaves. These phenolic components contribute to the plant’s natural resistance to pathogens and oxidative stress, while exhibiting antioxidant, anti-inflammatory, and antitumorigenic properties in vivo [4]. Despite this biochemical potential, most studies on *Quercus* species have focused on wood and bark extracts or on the valorization of forest by-products, leaving limited information on the dynamic responses of *Q. sideroxyla* leaves under controlled physiological stimuli.

Plant secondary metabolism is dynamically regulated by hormonal signaling pathways rather than being directly dependent of them. Among plant hormones, jasmonic acid (JA) and abscisic acid (ABA) are recognized as the main regulators of pathways related to defense and metabolic plasticity [5]. Jasmonates are frequently implicated in responses to wounds and pathogens, promoting the accumulation of phenolics, terpenoids, and alkaloids. In contrast, ABA acts as an integrative signal coordinating metabolic reallocation and developmental adjustment, modulating specialized metabolism beyond its classical role in abiotic stress responses [6,7]. Exogenous application of these hormones has been widely used as a stimulation strategy to trigger secondary metabolite biosynthesis in crops such as *Vitis vinifera* [8], *Camellia sinensis* [6,9], *Glycine max* [10,11], and *Arabidopsis thaliana* [12,13,14]. However, there is limited knowledge about the metabolic responses of *Quercus* species to phytohormonal elicitation, especially under controlled greenhouse conditions, where environmental variables can be standardized to isolate biochemical effects.

Metabolomic profiling provides a robust framework for elucidating the biochemical outcomes of hormonal treatments. The combination of liquid chromatography with tandem triple quadrupole mass spectrometry (LC-MS/MS) has become one of the most robust analytical tools for the specific quantification of phenolic compounds in complex plant matrices. This tool allows for the precise identification of structurally related metabolites, such as chlorogenic acid derivatives, quercetin glycosides, and procyanidin oligomers, which are often coeluted or masked in spectrophotometric assays [15]. Its sensitivity, accuracy, and reproducibility make it suitable for monitoring subtle metabolic variations induced by elicitors, even in small-scale experiments. Specific LC-MS/MS approaches allow for the detection of predefined sets of metabolites associated with specific biosynthetic pathways, providing a quantitative view of phenolic accumulation patterns.

Given the multivariate and highly correlated nature of phenolic datasets, the integration of machine-learning approaches such as Random Forest is particularly valuable, as it enables robust variable ranking, captures non-linear relationships among metabolites, and enhances the identification of discriminant compounds associated with specific hormonal treatments without relying on distributional assumptions.

Current research has demonstrated the effectiveness of LC–MS/MS for profiling phenolic compounds in oak-derived materials, particularly wood and bark extracts [16,17]. While several studies have also characterized phenolic composition in *Quercus* leaves, these analyses have generally been conducted without phytohormonal elicitation and have relied primarily on bulk phenolic indices or antioxidant capacity, rather than on the targeted resolution of hormone-responsive individual metabolites. Consequently, the metabolomic responses of oak leaves to exogenous phytohormonal elicitation remain largely unexplored, especially under controlled greenhouse conditions.

Studying juvenile *Quercus sideroxyla* leaves under controlled conditions, therefore, provides a valuable framework to characterize hormone-driven phenolic reprogramming without the confounding effects of environmental variability. This approach enables the identification of differential accumulation patterns among key phenolic classes, including hydroxycinnamic acids, flavonoid glycosides, and proanthocyanidins, during early developmental stages.

Given the long-lived and perennial nature of oaks, their regulatory mechanisms of secondary metabolism are expected to differ substantially from those described in annual model species. Therefore, resolving phytohormone-mediated phenolic regulation in *Q. sideroxyla* not only contributes to fundamental plant metabolic knowledge but also supports future applications in forest biotechnology, resilience strategies, and the valorization of leaf biomass. The use of validated LC–MS/MS workflows further ensure analytical robustness and reproducibility for comparative metabolomic studies within the genus *Quercus*.

Based on this framework, we hypothesized that (i) salicylic acid, jasmonic acid, and abscisic acid induce distinct and hormone-specific phenolic reprogramming patterns in juvenile *Q. sideroxyla* leaves; (ii) these responses exhibit differential temporal dynamics reflecting transient, adaptive, or stabilizing metabolic strategies; and (iii) multivariate and machine-learning-based analyses can resolve key discriminant metabolites underlying these hormone-dependent phenolic trajectories.

Therefore, the present study aimed to characterize the specific phenolic metabolomic profile of *Quercus sideroxyla* leaves following foliar application of salicylic acid, abscisic acid, and jasmonic acid under controlled greenhouse conditions using LC-MS/MS. This approach provides quantitative evidence of how different phenolic groups accumulate in response to hormonal elicitation, offering new insights into the modulation of secondary metabolism in oak seedlings and filling a key knowledge gap in phenolic metabolic profiles in oak leaves.

## 2. Results

### 2.1. Phenolic Profile

The metabolites present in the leaves of *Quercus sideroxyla* were identified using UPLC-ESI-MS/MS, which allowed for the accurate characterization of the phenolic compounds and hydrolyzable tannins modulated by the applied elicitors. Table 1 presents the complete list of detected compounds along with their abbreviated codes, retention times (RT), precursor ions, and MRM transitions used for structural confirmation. The metabolites are grouped into three main categories: phenolic acids, flavonoids, and hydrolyzable tannins.

It is important to note that although shikimic acid is not strictly classified as a phenolic acid, it is included in the table because of its central role as an intermediate in the shikimic acid pathway. This pathway is the starting point for the biosynthesis of phenylpropanoids, ellagitannins, and other secondary metabolites relevant to the elicited response. The detailed documentation of these compounds established a foundation for subsequent comparative analyses, enabling thorough evaluation of changes in relative abundance and accumulation dynamics triggered by SA, JA, and ABA at various concentrations and sampling times.

#### 2.1.1. Organic Acids and Conjugated Phenolic Acids

During the initial elution of compounds (RT ≈ 0.9–2.2 min), highly polar molecules such as quinic acid (QA), shikimic acid (ShA), and the first gallate sugars (GaHA (I), MGG (I)) are detected, which is consistent with their high hydroxylation and low hydrophobicity. Shikimic acid, while not strictly a phenolic acid, exhibits a comparable response to highly oxygenated carboxylic acids, which explains its early elution.

Hexosylated gallates (GaHA) are separated based on their elution properties. The GaHA (I) isomer (RT 1.06 min) elutes distinctly before the GaHA (II) isomer (RT 2.01 min). This indicates that the GaHA (I) isomer corresponds to a conformation or galloylation position that is more exposed to the solvent and more hydrophilic. In contrast, the GaHA (II) isomer is associated with a substitution position that favors hydrophobic interactions with the stationary phase. For example, substitution at C-6 of glucose versus more internal positions or a more compact conformation of the molecule.

In the case of galloylquinic acids (GaQA), a marked shift in retention times is observed: GaQA (I) (RT 1.50 min) elutes in the initial zone of the gradient, while GaQA (II) (RT 6.66 min) and GaQA (III) (RT 7.19 min) appear in a more organic region of the gradient. This separation indicates that GaQA (I) is a more polar regioisomer, meaning it is a galloylation at a position on the quinic acid that maintains greater accessibility of free hydroxyl groups. In contrast, GaQA (II) and (III) correspond to substitution positions that generate more hydrophobic structures or with greater π-π stacking capacity with the stationary phase. This pattern aligns with the observations made for other caffeoylquinic acids, where 3-, 4-, and 5-substituted isomers exhibit retention variations linked to alterations in the spatial arrangement of acyl and hydroxyl groups.

Overall, the progression of RT in quinic derivatives (QA, CQA, ChA, CryA, SQA, DCQA, GaQA I–III, CQA der) reflects a gradual increase in aromatic character and degree of substitution (monoacylated → diacylated), supporting the assignment of these peaks as conjugates of quinic acid with one or more caffeic or gallic groups in different positions.

#### 2.1.2. Flavonoids and Flavan-3-ol Derivatives

In the glycosylated flavonoid group, the hexosylated myricetin isomers MyH (I) (RT 6.19 min) and MyH (II) (RT 6.57 min) show partial separation, with retention times very close to each other. Given that both exhibit the same precursor ion (*m*/*z* 479.08) and identical main fragments (*m*/*z* 316.99, 179.02), this behavior is consistent with the presence of position isomers (e.g., myricetin-3-O-hexoside and myricetin-7-O-hexoside). These isomers differ in their structure, resulting in a slight variation in affinity for the stationary phase, but not in a differential fragmentation pattern. Alternatively, the contribution of anomeric isomers (α/β) with very similar behavior in reverse phase cannot be ruled out.

In contrast, the isomers of quercetin-O-β-glucuronide QβG (I) (RT 6.58 min) and QβG (II) (RT 7.41 min) demonstrate clear separation. The slight variation in RT, maintaining the same precursor ion (*m*/*z* 476.92) and characteristic fragments (*m*/*z* 300.99, 150.96), is consistent with position isomerism of the glucuronide bond, typically attributable to substitutions at C-3 or C-7 of the flavonoid skeleton. The isomer that elutes first (QβG (I)) is presumed to be the most polar (i.e., greater exposure of free hydroxyl groups or lower stacking capacity), while QβG (II) corresponds to a more hydrophobic arrangement or one with greater affinity for the stationary phase.

Other flavonoids, including quercetin 3-O-glucoside (QG), kaempferol 3-O-glucoside (KG), kaempferol rutinoside (KRu), hesperidin (H), and quercetin rhamnoside (QRh), are found in the middle-late zone of the gradient (RT ≈ 6.3–8.6 min). This is consistent with the increase in mass, number of aromatic rings, and substitutions with deoxy sugars (rhamnose). These characteristics contribute to a balance between polarity (sugars) and hydrophobicity (flavonoid nucleus), which is consistent with the observed retention times.

Within this flavonoid fraction, flavan-3-ol oligomers were also detected, including procyanidin B1 (PB1) and a procyanidin derivative, which eluted in the middle region of the gradient. These compounds represent condensed tannins derived from catechin/epicatechin units and are structurally and biosynthetically distinct from hydrolyzable tannins based on galloyl or HHDP motifs. Their inclusion within the flavonoid group reflects their origin from the flavan-3-ol pathway rather than from galloylated glucose cores.

#### 2.1.3. *Hydrolyzable Tannins*

In the fraction of hydrolyzable tannins, the series of galloylated glycosides exhibits a particularly informative chromatographic pattern. The monogalloyl glucose MGG (I), (II), and (III), with retention times of 1.05, 1.32, and 1.96 min, respectively, elute in the most polar region of the gradient, although with a progressive increase in retention time. This distribution is consistent with the presence of positional isomers in the glycosidic skeleton. Substitutions at C-2 or C-3 generate molecules that are more exposed to the solvent and, therefore, more hydrophilic. Meanwhile, galloylation at C-4 or C-6 favors more compact conformations with greater hydrophobic interaction with the stationary phase, which explains the later eluents. Similarly, digalloyl glucose (DGG) displays multiple peaks with retention times of 1.56, 2.04, and 3.92 min, indicating the presence of at least three distinct structural configurations, including 2,3-; 2,6-; and 3,6-di-O-galloyl glucose patterns. The shift toward later retention times as the degree of galloylation increases is consistent with the increase in aromatic character and the possibility of additional π-π stacking.

Trigalloyl glucose (TGG) (I), (II), and (III) demonstrate even wider dispersion in the chromatogram, with retention times of 1.74, 4.38, and 9.21 min, respectively. Specifically, the TGG isomer (III), which is observed at the end of the gradient, likely corresponds to a structural arrangement that confers a more hydrophobic and stacked conformation. This enhances the interaction with the stationary phase, explaining its significant retention time.

Derivatives with HHDP units (HHDPGG, HHDPmGG, HHDPdGG) and ellagic acid glycosides are in an intermediate region of the gradient (approximately between 1.0 and 6.0 min), showing retention times higher than those of mono- and digalloyl glucose of comparable mass. The presence of the hexahydroxydiphenyl (HHDP) group, along with its lactonization to form the ellagic acid nucleus, provides an aromatic bicyclic system with high hydrophobic interaction and stacking capacity, which explains the higher retention observed. Within each series—HHDPGG (I–III) and HHDPdGG (I–III)—the increase in retention times can be attributed to variations in the position of the additional galloyl groups. These variations alter the exposure of hydroxyl groups and modulate the conformational compaction of the tannin.

Finally, ellagic acid derivatives (EAP, EAG I–II, EAX I–II, EAR I–II, MPEA, and EA) follow a pattern consistent with the polarity of the linked sugar. Pentosides and xylosides tend to elute slightly earlier than rhamnosides, while the aglycone ellagic acid has an intermediate retention time. This is due to the balance between its compact polyphenolic nature and the absence of hydrophilic sugars that could increase its polarity and decrease its retention time.

From a structural point of view, these patterns allow us to hypothesize about the nature of the conjugations (i.e., position of galloylation, type of sugar, degree of substitution) and support the assignment of the isomeric peaks as regio-isomeric variants of the same base entities. However, definitive confirmation of the position of the substituents requires further study. Therefore, the proposals derived from the retention times should be considered plausible but still provisional in the context of this work.

### 2.2. Phytohormone-Induced Modulation of Phenolic Metabolism

**Figure 1:**
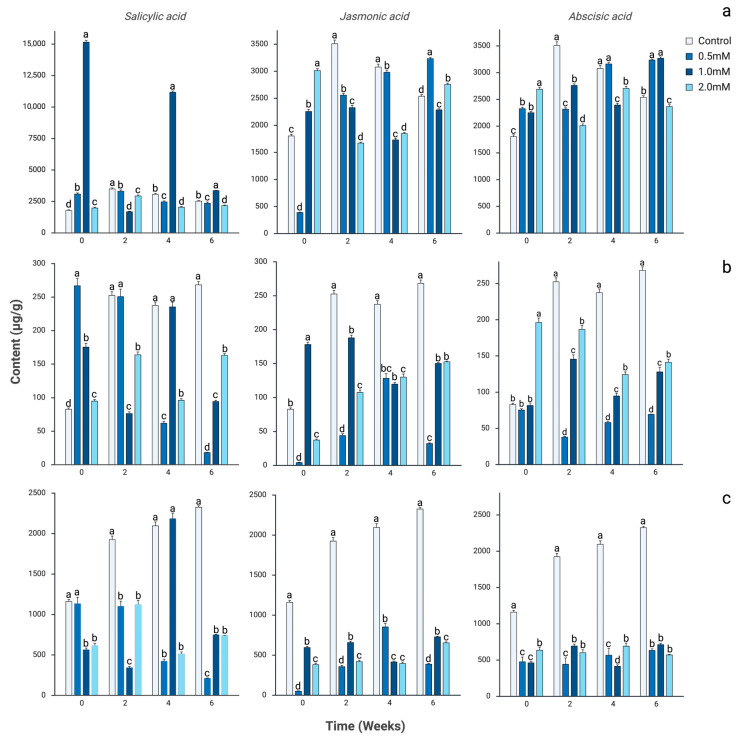
Total accumulation of (**a**) phenolic acids, (**b**) flavonoids, and (**c**) hydrolyzable tannins identified by UPLC-ESI-MS/MS in leaves of *Quercus sideroxyla* treated with salicylic acid, jasmonic acid, and abscisic acid for six weeks. Values are expressed as mean ± standard deviation. Data were analyzed using two-way ANOVA (time × treatment), followed by Bonferroni post hoc test. Different letters indicate statistically significant differences among treatments within the same time point (*p* < 0.05).

Figure 1 presents the total accumulation of phenolic acids, flavonoids, and hydrolyzable tannins in *Quercus sideroxyla* subjected to foliar applications of salicylic acid (SA), jasmonic acid (JA), and abscisic acid (ABA) over a six-week period. In Figure 1a, corresponding to phenolic acids, the effect of SA at 1.0 mM is particularly notable, inducing an 8.4-fold increase compared with the control—the highest response observed among all treatments. Although this hyperaccumulation declines by week 2, levels rise again by week 4, remaining 3.6-fold above the control. By week 6, the values converge with those recorded under JA and ABA, revealing a pulsatile and transient response, characterized by an early peak, subsequent attenuation, and a mid-term reactivation.

In contrast, JA elicits a more stable and gradually increasing profile, without pronounced peaks. The 0.5 and 2.0 mM treatments exhibit consistent increases toward the later weeks, reflecting a sustained accumulation pattern. ABA displays a similarly progressive and upward trajectory, with gradual increments across all concentrations and maximum levels reached at 0.5 and 1.0 mM by week 6, suggesting a continuous and less oscillatory activation compared with the response induced by SA.

The accumulation of flavonoids (Figure 1b) exhibits changes of lower absolute magnitude than those observed for phenolic acids; however, the quantitative patterns clearly distinguish the metabolic influence of each elicitor. At week 0, SA at 0.5 mM induces a 3.2-fold increase relative to the control, whereas the 1.0 mM concentration produces only a 2.1-fold rise and 2.0 mM causes a modest 1.1-fold increase. By week 2, SA 1.0 mM shows a marked reduction (0.30-fold vs. control), evidencing a strong suppressive effect before recovering at week 4 (~1.0-fold of the control) and stabilizing by week 6 at ~0.35-fold. JA, by contrast, produces a steady and moderate accumulation: at week 0 the 1.0 mM treatment increases flavonoids by 2.1-fold, and throughout the six weeks remains within a controlled range of 0.5–1.6-fold relative to its time-matched controls. ABA displays the most predictable pattern, with progressive increases across all concentrations; notably, 2.0 mM reaches 1.5–1.8-fold above the control at weeks 2–6, indicating sustained stimulation of flavonoid biosynthesis.

Figure 1c reveals sharper quantitative contrasts in hydrolyzable tannins. SA generates a pronounced early inhibition, particularly at 1.0 mM, where levels drop to 0.47-fold of the control at week 0. However, at week 4 this same concentration exhibits a rebound to 1.04-fold, the only case of transient overstimulation observed in this metabolite group. JA begins at extremely low values (week 0 levels at 0.04-fold of the control for 0.5 mM) but progressively recovers until reaching 0.32–0.34-fold of the control by week 6. ABA induces the most robust and stable response: at 1.0 mM, hydrolyzable tannins reach 0.59-fold at week 0, increase to 0.36-fold at week 2, and peak at 0.30–0.31-fold above the control in weeks 4–6, reflecting a sustained and cumulative activation pattern.

Together, these quantitative trends indicate that each phytohormonal treatment imprints a distinct temporal and metabolic trajectory. SA acts as a strong but transient activator, characterized by sharp pulses of overaccumulation followed by rapid attenuation. JA induces controlled, moderate, and progressively stabilizing increases. ABA promotes continuous and concentration-dependent accumulation, generating the most persistent enhancement across phenolic classes. These data highlight the differential regulatory roles of phytohormones and provide a quantitative foundation for selecting elicitation strategies tailored to specific phenolic families.

### 2.3. Multivariate Analysis of Phenolic Signatures Under SA, JA, and ABA Elicitation

The PLS-DA analysis with discrete recoding of each elicitor-concentration-week combination (e.g., SA0.5mM_2, Control_6) allowed for an integrated assessment of phenolic reprogramming across the four experimental stages. The optimal three-component model explained a substantial proportion of metabolomic variability (R^2^ = 0.648) and demonstrated acceptable predictive ability, despite the large number of classes (Q^2^ = 0.561). Permutation testing (1000 permutations) corroborated statistical significance, with significant values for prediction accuracy (*p* = 0.001) and distance between groups (*p* < 0.001). This confirms that the observed separation is not a product of chance.

**Figure 2:**
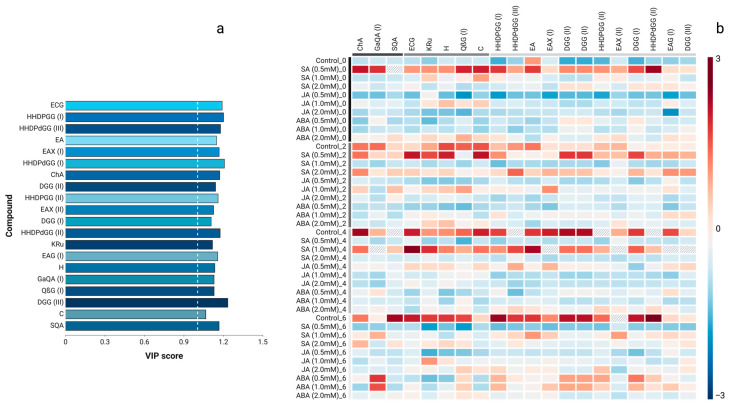
Variable Importance in Projection (VIP) scores of discriminant phenolic metabolites (VIP ≥ 1.0) identified by PLS-DA (**a**), and heatmap of their relative accumulation patterns across treatments (**b**). In panel (**b**), colors represent standardized abundance (z-scores), with red and blue indicating higher and lower levels relative to the mean, respectively. Dotted white line indicates VIP score > 1.0. Compound codes correspond to those listed in Table 1.

The VIP scores and heatmap demonstrate that ellagitannin derivatives, HHDPdGG, DGG, and flavan-3-ols dominate overall discrimination (Figure 2a). Salicylic acid exhibited the most intense and consistent accumulation, with notable responses in SA0.5mM_0, SA0.5–2.0mM_2, and SA1.0mM_4, surpassing JA and ABA by a large margin (Figure 2b).

### 2.4. Hormone-Dependent Hierarchies and Integrative Roles of Discriminant Phenolic Metabolites

Table 2 integrates two complementary approaches to identify discriminating metabolites induced by phytohormones in young leaves: (i) MANOVA, which quantifies the proportion of variance explained by time, treatment, and their interaction (partial η^2^), and (ii) Random Forest (RF), which ranks the contribution of each metabolite to the classification using Mean Decrease Accuracy (MDA). Together, the results confirm that the hormonal effect highly structures the phenolic response and that the selected metabolites are organized into three dominant chemical axes: phenolic acids, flavonoids, and hydrolyzable tannins, with specific integrative roles.

In the SA treatment, a particularly intense and consistent reprogramming signal was observed: the selected markers present a partial η^2^ that is practically unitary for the three effects (≈0.99–1.00), indicating an almost deterministic dependence on time and dose. In phenolic acids, QA reached the maximum value of importance in RF (Maximum; MDA = 0.171) and acted as the central node of the quinic scaffold, supporting the reorganization of phenylpropanoid-derived conjugates. Consistently, GAHA (I) and GaQA (II) reflected early transitions and diversion of flux from quinic acid to secondary phenols (High and Medium-high categories, respectively), while ChA indicated redirection toward hydroxycinnamic acids (Medium-high). In flavonoids, KRu and QRh showed high sensitivity to SA (Medium-high and High), suggesting fine-tuning of conjugation and plasticity of the quercetin nucleus. In hydrolyzable tannins, EAG (II) and EAR (II) stood out as structural biomarkers of the ellagic axis (both Very high), complemented by MPEA (High) as an indicator of terminal modulation of ellagic derivatives and by DGG (II) (Medium) as a rearrangement of the galloyl pool.

In JA, the pattern was equally robust in MANOVA, but with an RF importance hierarchy that more clearly separates “background nodes” from “primary discriminants.” In phenolic acids, DCQA (Medium-high; MDA = 0.045) and SQA (Medium-high; MDA = 0.033) reflected a fine modulation of the phenylpropanoid flux and the shikimate/quinic node, respectively, while ChA, QA, and CaH (all Medium) functioned as contextual indicators of reconfiguration of the quinic and hydroxycinnamic acceptor pool. In flavonoids, a distinctive JA signature emerged: QG was classified as Very high (MDA = 0.110), indicating strong channeling towards flavonol glycosylation; C (High; MDA = 0.098) acted as a structural bridge towards polymerization pathways; KRu (High; MDA = 0.055) and QβG (I) (Medium-high; MDA = 0.039) complemented the evidence of stabilization by conjugation. In hydrolyzable tannins, MPEA was the main discriminant (Maximum; MDA = 0.138), followed by EAR (II) (Very high; MDA = 0.126), which supports a redirection towards advanced ellagic metabolism under JA. Additionally, HHDPdGG (II) (Medium-high; MDA = 0.044) and MGG (II) (Medium-high; MDA = 0.034) showed remodeling of the galloyl/ellagitannin pool towards more complex structures; at this point, its relevance is interpreted as part of a JA-dependent “tannin remodeling module.”

For ABA, the sustained and highly discriminating signal revealed by the MANOVA–RF integration suggests a regulatory role centered on the reorganization of phenolic precursors and on the coordination of the proanthocyanidin–flavonoid axis, together with ellagic-related structures. This pattern is consistent with a hormone-driven adjustment of phenolic homeostasis rather than a transient stress-induced response. In phenolic acids, SQA was the dominant marker (Very high; MDA = 0.121) and was interpreted as rerouting towards synapoylquinic conjugates and defensive structural remodeling; GaHA (I) (Medium-high; MDA = 0.032) suggested a “gatekeeper” of the gallate pool, while ChA and DCQA (both Medium) captured the rebalancing of caffeoylquinic esters and time × dose windows for diester accumulation. In flavonoids, PB1 (procyanidin B1) was the primary discriminant (Maximum; MDA = 0.198) and, as it derives from flavan-3-ols (not ellagitannins), it reorients the conclusion towards a marked activation of the proanthocyanidin axis under ABA (dimerization/condensation). QG (High; MDA = 0.074) and KRu (High; MDA = 0.051) supported robust control of flavonol conjugation, while ECG (Medium; MDA = 0.023) suggested adjustment of galloylated flavanols associated with antioxidant structuring. In hydrolyzable tannins, EAR (II) remained an indicator of ellagic maturation (Very high), with MPEA (Very high; MDA = 0.114) as a sensitive integrator of the HHDP–ellagic axis and EAR (I) (Medium) as a support for the basal state through time × dose interaction.

In summary, the table shows that SA prioritizes central nodes of the quinic skeleton and the ellagic axis with a highly deterministic signature; JA combines a marked flavonoid signal (QG/C/KRu) with activation of terminal ellagic derivatives (MPEA/EAR) and galloyl remodeling; while ABA is distinguished by a strong discriminating capacity in proanthocyanidins (PB1) and a sustained reconfiguration of quinic conjugates and flavonoids, coexisting with a persistent ellagic component.

## 3. Discussion

The present study provides an integrated view of hormone-specific phenolic reprogramming in *Q. sideroxyla* juvenile leaves, combining targeted metabolite profiling with multivariate statistics and machine-learning–based feature ranking to resolve distinct signatures associated with SA, JA, and ABA elicitation. Oak leaf extracts obtained with aqueous acetone revealed a broad coverage of the phenylpropanoid–flavonoid–ellagitannin continuum, reflecting the intrinsic metabolic complexity of *Quercus* leaves and providing a robust basis for resolving hormone-dependent reprogramming patterns rather than simple compositional differences. Beyond compositional coverage, the integration of MANOVA (time, treatment, and interaction effects), PLS-DA, and Random Forest (MDA-based importance) revealed that each hormonal treatment induced a coherent yet differentiated pattern of phenolic modulation, reflected in the selective contribution of specific metabolites across the shikimate–phenylpropanoid–flavonoid–ellagitannin continuum (Table 2). These results directly address the working hypothesis of this study, namely that exogenous application of distinct phytohormones would induce hormone-specific and time-dependent phenolic reprogramming in juvenile *Q. sideroxyla* leaves, resulting in differentiated accumulation trajectories across major phenolic classes under controlled greenhouse conditions.

The detection of acylated quinic acid derivatives, glycosylated flavonoids, and hydrolyzable tannins in *Q. sideroxyla* is consistent with their biosynthetic origin in the shikimate–phenylpropanoid pathway, a central metabolic route extensively characterized in plants [18,19]. Previous studies have demonstrated that variation in the availability of intermediates such as shikimate and quinate can condition the formation of hydroxycinnamic esters, including chlorogenic acid and structurally related derivatives [20]. In this context, the identification of multiple caffeoylquinic and dicaffeoylquinic acids in *Q. sideroxyla* is compatible with the established biochemical roles of BAHD acyltransferases involved in hydroxycinnamoyl ester formation, commonly referred to as hydroxycinnamoyl-CoA: quinate hydroxycinnamoyl transferase (HQT) and hydroxycinnamoyl-CoA: shikimate/quinate hydroxycinnamoyl transferase (HCT), which catalyze the esterification of hydroxycinnamic acids onto quinate or shikimate acceptors [21,22]. However, interpretations are constrained to coordinated changes in metabolite abundance rather than direct enzymatic regulation, as the present study does not include measurements of metabolic flux, enzyme activity, or gene expression. Accordingly, the chemical landscape summarized in Table 2 should be viewed as a structured framework for future integrative validation using transcriptomics, enzymatic assays, or isotopic labeling in *Q. sideroxyla*.

Hormone-specific differences in phenolic accumulation further highlight distinct regulatory modes for SA, JA, and ABA. Importantly, the pulse-based sampling design, with measurements every two weeks, enabled discrimination between transient, adaptive, and stabilizing response patterns that would likely remain unresolved in single-time-point experimental approaches. Under SA elicitation, the response displayed a pronounced pulsatile behavior, consistent with the role of SA as a primary immune signal capable of triggering rapid but energetically demanding metabolic reprogramming [23,24]. In agreement with this pattern, Table 2 shows that SA discrimination was driven by metabolites associated with central precursor availability and ellagic-related structural states, supporting a strategy of metabolic priming rather than sustained investment.

In contrast, JA elicitation promoted a more gradual and stable modulation of phenolic pools, consistent with its role in adaptive defenses against herbivory, mechanical damage, and necrotrophic pathogens [25,26]. The absence of sharp accumulation peaks suggests that *Q. sideroxyla* responds to JA through sustained metabolic adjustments, likely optimizing carbon allocation toward structural reinforcement and long-term protection. This interpretation is supported by the convergence between MANOVA effect sizes and Random Forest rankings, which consistently identified advanced caffeoylquinic esters, flavonol conjugates, and condensed ellagitannins as discriminant markers of JA elicitation (Table 2). Notably, Random Forest was used strictly as a feature-ranking approach, highlighting variables that best discriminate hormonal conditions without implying causal regulatory relationships.

ABA elicitation resulted in a progressive and sustained accumulation across phenolic acids, flavonoids, and hydrolyzable tannins. Because plants were maintained under controlled, non-stressful greenhouse conditions, this response is interpreted as ABA-mediated metabolic reprogramming rather than a direct stress response. ABA is known to function as an integrator of developmental and metabolic signaling, modulating specialized metabolism through core regulators such as SnRK2 kinases and ABF/AREB transcription factors even in the absence of water deficit [25,27,28].

Consistently, ABA-treated plants exhibited a discriminant profile in which flavonoid- and proanthocyanidin-associated markers, together with ellagic derivatives, contributed strongly to group separation (Table 2), indicating stabilization of phenolic output with reduced temporal volatility relative to SA and a broader engagement of antioxidant and structural phenolics.

Taken together, these results demonstrate that *Q. sideroxyla* not only discriminates among SA, JA, and ABA signals but translates each of them into distinct phenolic phenotypes characterized by different balances between metabolic plasticity, structural investment, and homeostatic regulation. The combined use of time-resolved sampling, MANOVA, and machine-learning–based feature ranking strengthens confidence in the robustness of these hormone-specific signatures. Although the present evidence is limited to abundance-based phenotyping, the concordance between statistical effect sizes and Random Forest importance values (Table 2) provides a solid analytical basis for defining hormone-guided elicitation targets in oak leaves and for prioritizing mechanistic studies aimed at uncovering the regulatory architecture underlying phenolic metabolism.

## 4. Materials and Methods

### 4.1. Plant Material and Greenhouse Conditions

Juvenile plants of *Quercus sideroxyla* Bonpl. were used in this study. The plant material originated from acorns collected from naturally growing adult trees in the El Salto region, Pueblo Nuevo, Durango, Mexico (23°46′53.29″ N; 105°21′19.93″ W; approximately 2500 m above sea level). Acorns were collected during the natural fruiting season and selected based on visual integrity and absence of physical damage or fungal infection.

Acorn germination and seedling establishment were carried out following the protocol described by Maldonado Aguilar [29], which includes surface cleaning, controlled germination, and early stage seedling management to ensure uniform plant development. The resulting seedlings were grown under nursery conditions until establishment. Juvenile plants were kindly provided by the National Institute of Forestry, Agricultural and Livestock Research (INIFAP), Guadiana Valley Experimental Center (Durango, Mexico).

At the beginning of the phytohormone treatments, plants were approximately 10 months old and displayed fully expanded leaves and a consolidated root system, corresponding to an early juvenile developmental stage typical of tree species. Only morphologically uniform plants were selected for the experiment in order to minimize variability related to growth stage.

After transfer to the greenhouse facilities of the Conahcyt National Laboratory for the Evaluation of Biotic Products (LaNAEPBi), plants were maintained under controlled environmental conditions, including a 12 h light/12 h dark photoperiod, a constant temperature of 22 ± 2 °C, and drip irrigation. Each plant was cultivated individually in a 3 L black polyethylene bag containing a standardized organic–inorganic substrate, composed of a peat-based organic fraction and perlite in a 2:1 (*v*/*v*) ratio, providing adequate aeration, drainage, and water-holding capacity for juvenile tree growth, and all plants were managed under identical horticultural conditions throughout the experimental period.

### 4.2. Experimental Design and Treatments with Phytohormone Elicitors

The concentrations of salicylic acid, jasmonic acid, and abscisic acid were selected based on their widespread use in elicitation studies aimed at inducing metabolic and defense-related responses in plant tissues under controlled conditions [30]. Comparable concentration ranges have been previously employed in foliar elicitation experiments to trigger phenylpropanoid-related responses without causing visible phytotoxic symptoms.

The study was designed using a randomized complete block design, with 10 juvenile plants per treatment. Three phytohormonal elicitors were evaluated: SA, JA, and ABA, each at concentrations of 0.5, 1.0, and 2.0 mM. The solutions were prepared in 10% (*v*/*v*) aqueous ethanol, used as a vehicle. Applications were made by means of fine foliar spraying until uniform coverage of the foliage and incipient runoff were achieved.

The elicitation scheme consisted of periodic applications every 15 days, with samples collected systematically 24 h after each application. The first spray was applied at time zero, and 24 h later, the first sampling was performed to evaluate the early response induced by the elicitor. Subsequently, the plants remained untreated for 15 days, after which a second application was made, followed again by sampling at 24 h. The same procedure was repeated on days 30 and 45 of the experiment, completing a total of four elicitation cycles and four sampling points, always within the 24-h early response window.

This design allows for consistent evaluation of the acute response (24 h) induced by each elicitor, while incorporating the cumulative effect of repeated applications over time. This is methodologically relevant for exploring physiological adjustments associated with recurrent hormonal exposures. Throughout the experiment, a control group was maintained that received only the vehicle solution (10% *v*/*v* aqueous ethanol, without phytohormones) under the same spraying conditions and schedule.

### 4.3. Sampling, Conservation, and Preparation of Plant Material

The leaves were collected from the middle portion of the canopy of each juvenile plant, selecting fully expanded and physiologically mature leaves. The incision was executed at a 45° angle using sterilized scissors. The collected material was immediately immersed in liquid nitrogen to slow metabolic activity and then stored at −84 °C until processing.

The frozen samples were freeze-dried for 48 h and stabilized in desiccators. The dried leaves were ground in an IKA MF 10 mill (IKA Works, Wilmington, NC, USA) equipped with a #100 mesh, producing a homogeneous powder suitable for extraction.

### 4.4. Extraction of Phenolic Compounds

Phenolic compounds were extracted following an aqueous acetone-based protocol adapted from previously published studies on *Quercus leaves* and other woody plant matrices [31]. Briefly, for each sample, 20 mg of powder (particle size of 149 µm) was weighed, and 1 mL of 70% (*v*/*v*) aqueous acetone was added. The samples were then homogenized for one minute at 20,000 rpm (IKA Works, Wilmington, NC, USA), followed by four 15-s sonication cycles with 45-s intervals (Branson Ultrasonics, Brookfield, CT, USA). Following centrifugation at 15,000×*g* for 10 min at 4 °C in a Heraeus Labofuge 400R centrifuge (Kendro Laboratory Products, Asheville, NC, USA), the resulting clear layer was collected. The collected layer was then evaporated to dryness using a CentriVap system (Labconco, Kansas City, MO, USA). Finally, the samples were resuspended in 500 µL of HPLC-grade methanol. Finally, the sample was subjected to a second round of centrifugation under the same conditions, and a 100 µL aliquot was retrieved for analysis by liquid chromatography coupled with mass spectrometry.

### 4.5. Analysis of Phenolic Compounds by UPLC-ESI-MS/MS

Phenolic compound analysis was performed using ultra-high-performance liquid chromatography coupled with tandem mass spectrometry (UPLC-ESI-MS/MS) using an Acquity UPLC system (Waters Corp., Milford, MA, USA) coupled to a Xevo TQ-S triple quadrupole mass spectrometer (Waters Corp.). Chromatographic separation was performed on an Acquity UPLC BEH C8 column (50 mm × 2.1 mm, 1.7 µm), using 7.5 mM formic acid-acidified water as mobile phase A and acetonitrile as mobile phase B. The flow rate was maintained at 0.250 mL/min, and a volume of 1 µL was injected. The elution gradient consisted of an initial phase of 95% A, followed by a progressive decrease to 0% A between 6.7 and 8.0 min; subsequently, a wash with 100% B was maintained, and finally, the initial conditions were reestablished for column re-equilibration. The chromatographic gradient and separation conditions were established according to the targeted phenolic profiling strategy described by García-Villalba et al. [32].

The spectrometry system operated in negative ionization mode (ESI−). Source parameters included a capillary voltage of 1.5 kV, a source temperature of 150 °C, and a desolvation temperature of 350 °C. The desolvation gas flow was maintained between 646 and 650 L/h, the cone gas flow at 150 L/h, and the nebulizer gas at 7 bar. The collision gas flowed at approximately 0.24–0.25 mL/min. These conditions allowed for stable and efficient ionization of the phenolic acids, flavonoids, and ellagitannins present in the extracts.

Data acquisition was performed using multiple reaction monitoring (MRM) functions specific to the precursor-product transitions corresponding to hydroxybenzoic acids, hydroxycinnamic acids, flavonoids, and monomeric and oligomeric ellagitannins. Collision energies were set between 2 and 20 eV, depending on the precursor mass and the expected fragmentation for each compound. Additionally, an MS/MS mode with enhanced scanning in the *m*/*z* 300–1100 range was used to confirm the identity of the compounds by characteristic fragmentation patterns. All data acquisition and processing were performed using MassLynx 4.1 software and the TargetLynx module (Waters Corp.), under standardized integration parameters to ensure consistency in quantification.

Metabolite identification was based on matching retention times, precursor masses, MRM transitions, and fragmentation with analytical standards and specialized literature. Quantification was performed using external calibration curves prepared with phenolic acid, flavonoid, and flavan-3-ol standards. In cases where specific commercial standards were not available, quantification was performed putatively using structurally related standards, which allowed the results to be expressed as equivalents of the compound used as a reference. This approach is widely accepted for targeted metabolomics of ellagitannins and complex phenolic compounds.

### 4.6. Statistical Analysis

Prior to statistical analysis, the data underwent a quality control process. This process verified the consistency of the values, detected obvious outliers, and evaluated modeling assumptions. When relevant to meet the assumptions of the parametric models, some variables were transformed to stabilize the variance and approximate normality. The effects of hormonal treatments were evaluated using a factorial MANOVA in IBM SPSS Statistics v29, considering time, treatment, and their interaction as factors. When significant effects were identified, multiple comparisons were adjusted using the Bonferroni correction (*p* < 0.05). For multivariate analysis, the data were processed in MetaboAnalyst v6.0 using auto-scaling (mean-centered and divided by standard deviation), equivalent to transformation to z-scores, without applying additional normalization or transformation steps within the platform. PLS-DA was used to differentiate between hormonal conditions and identify compounds with the greatest discriminating contribution. The performance of the model was evaluated using the R^2^ and Q^2^ metrics. Additionally, Random Forest was employed to prioritize metabolites based on Mean Decrease in Accuracy (MDA), facilitating a comprehensive selection of biomarkers.

## 5. Conclusions

This study demonstrates that *Q. sideroxyla* exhibits hormone-specific phenolic reprogramming trajectories in response to SA, JA, and ABA elicitation. By integrating time-resolved sampling with multivariate and machine-learning analyses, we show that SA induces transient, pulsatile phenolic responses consistent with metabolic priming, JA promotes sustained adaptive modulation linked to structural and defensive phenolics, and ABA stabilizes phenolic output through homeostatic regulation.

Within the experimental framework applied here, distinct temporal windows and concentration ranges were associated with maximal hormonal differentiation. SA elicitation produced its most pronounced effects at early sampling points (24 h after application), particularly at intermediate concentrations, highlighting its role in rapid metabolic priming. In contrast, JA-driven phenolic modulation became more evident after repeated applications, with stronger discrimination observed at later developmental stages of juvenile leaves. ABA treatments promoted progressively stabilized phenolic accumulation across sampling points, indicating a cumulative regulatory effect rather than a transient response.

Importantly, these responses were observed under non-stressful conditions, supporting the view that plant hormones actively regulate specialized metabolism rather than merely amplifying stress responses. While interpretations are constrained to coordinated changes in metabolite abundance, the phenolic signatures identified here provide a robust framework for future integrative studies incorporating transcriptomics, enzymatic assays, or isotopic labeling.

Overall, this work establishes a conceptual and analytical basis for hormone-guided modulation of phenolic metabolism in woody plants, with implications for plant physiology, metabolic engineering, and the development of phenolic-rich functional ingredients.

## Figures and Tables

**Table 1:** List of phenolic acids, flavonoids, and hydrolyzable tannins identified by UPLC-ESI-MS/MS, with their corresponding analytical parameters.

No.	Compound	Code	RT (min)	Parent Ion	Main Transitions
1.	Caffeic acid hexoside	CaH	0.95	341.08	179.05, 135.08
2.	Quinic acid	QA	1.00	191.20	93.06, 85.06
3.	Shikimic acid *	ShA	1.02	173.18	111.07, 93.06
4.	Galloyl acid hexoside iso I	GaHA (I)	1.06	331.06	169.01
5.	Galloylquinic acid iso I	GaQA (I)	1.50	343.06	191.05, 169.05
6.	Gallic acid	GA	1.61	169.15	125.05, 79.07
7.	Caffeoylquinic acid	CQA	1.77	353.10	191.20, 85.10
8.	Galloyl acid hexoside iso II	GaHA (II)	2.01	331.06	169.01
9.	Dihydroxybenzoic acid hexoside	DHBAH	2.20	315.07	152.04
10.	p-Coumaroyl tartaric acid	TCA	4.33	295.04	163.02, 118.92
11.	Chlorogenic acid	ChA	4.37	353.10	191.20, 85.10
12.	Cryptochlorogenic acid	CryA	5.14	353.10	191.20, 85.10
13.	Sinapoylquinic acid	SQA	5.24	397.11	223.01, 191.07
14.	Dicaffeoylquinic acid	DCQA	6.65	515.00	353.10, 191.20
15.	Galloylquinic acid iso II	GaQA (II)	6.66	343.06	191.05, 169.05
16.	Galloylquinic acid iso III	GaQA (III)	7.19	343.06	191.05, 169.05
17.	Caffeoylquinic acid derivative	CQA der	11.32	353.10	191.20, 85.10
18.	Procyanidin B1	PB1	3.82	577.44	407.21, 289.18
19.	Catechin	C	4.39	289.00	245.06, 203.00, 122.96
20.	Epicatechin gallate	ECG	4.60	441.27	169.04
21.	Myricetin hexoside (I)	MyH (I)	6.19	479.08	316.99, 179.02
22.	Rutin	R	6.38	609.04	270.94, 254.89
23.	Myricetin hexoside (II)	MyH (II)	6.57	479.08	316.99, 179.02
24.	Quercetin 3-O-β-glucuronide (I)	QβG (I)	6.58	476.92	300.99, 150.96
25.	Quercetin 3-O-glucoside	QG	6.61	463.36	300.42, 271.11, 151.00
26.	Kaempferol 3-O-glucoside	KG	7.30	447.30	284.24, 255.12
27.	Quercetin 3-O-β-glucuronide (II)	QβG (II)	7.41	476.92	300.99, 150.96
28.	Procyanidin derivative	P-der	7.84	577.44	407.21, 289.18
29.	Hesperidin-like flavanone	H	8.26	609.04	270.94, 254.89
30.	Quercetin rhamnoside	QRh	8.35	447.09	301.03, 178.99, 151.00
31.	Kaempferol rutinoside	Kru	8.57	593.15	285.03
32.	Quercetin	Q	8.65	300.95	178.91, 150.96
33.	HHDP-monogalloyl glucose	HHDPmGG	1.05	481	301, 169
34.	Monogalloyl glucose (I)	MGG (I)	1.05	331	271, 241, 169
35.	Monogalloyl glucose (II)	MGG (II)	1.32	331	271, 241, 169
35.	HHDP-galloyl-glucose (I)	HHDPGG (I)	1.52	633	593, 481, 301
37.	Digalloyl glucose (I)	DGG (I)	1.56	483	331, 313, 169
38.	Trigalloyl glucose (I)	TGG (I)	1.74	635	465, 313
39.	HHDP-galloyl-glucose (II)	HHDPGG (II)	1.87	633	593, 481, 301
40.	Monogalloyl glucose (III)	MGG (III)	1.96	331	271, 241, 169
41.	Digalloyl glucose (II)	DGG (II)	2.04	483	331, 313, 169
42.	HHDP-galloyl-glucose (III)	HHDPGG (III)	3.71	633	593, 481, 301
43.	Digalloyl glucose (III)	DGG (III)	3.92	483	331, 313, 169
44.	HHDP-di-galloyl-glucose (I)	HHDPdGG (I)	4.19	785	748, 633, 615, 483, 301
45.	Ellagic acid pentoside	EAP	4.36	433	301
46.	Trigalloyl glucose (II)	TGG (II)	4.38	635	465, 313
47.	Ellagic acid glucose (I)	EAG (I)	4.82	463	300, 113
48.	HHDP-di-galloyl-glucose (II)	HHDPdGG (II)	4.85	785	748, 633, 615, 483, 301
49.	HHDP-di-galloyl-glucose (III)	HHDPdGG (III)	5.37	785	748, 633, 615, 483, 301
50.	Ellagic acid xyloside (I)	EAX (I)	5.63	433	301, 257
51.	Ellagic acid glucose (II)	EAG (II)	5.97	463	300, 113
52.	Ellagic acid rhamnoside (I)	EAR (I)	6.00	447	300, 257
53.	Pentagalloylglucose (I)	PGG (I)	6.14	939	787, 769, 717
54.	Ellagic acid xyloside (II)	EAX (II)	6.19	433	301, 257
55.	Ellagic acid	EA	6.23	301	284, 229, 145
56.	Methylated pentoside ellagic acid	MPEA	6.29	447	299, 113
57.	Pentagalloylglucose (II)	PGG (II)	6.67	939	787, 769, 717
58.	Ellagic acid rhamnoside (II)	EAR (II)	6.96	447	300, 257
59.	Trigalloyl glucose (III)	TGG (III)	9.21	635	465, 313

* Shikimic acid is included because of its importance in the shikimic acid pathway as a precursor of phenolic compounds, although it is not strictly classified as a phenolic acid. HHDP denotes a hexahydroxydiphenoyl group.

**Table 2:** Integration of MANOVA and Random Forest (RF) for the identification of discriminating biomarkers induced by phytohormones.

Chemical Class	Compound	MANOVA			RF Category *	Integrative Role
		Time	Treatment	Interaction		
***Salicylic acid***						
*Phenolic acids*	QA	1.000	1.000	1.000	Maximum (0.171)	Central metabolic node; primary biomarker of SA-driven phenylpropanoid reprogramming
	GAHA (I)	0.999	0.998	1.000	High (0.054)	Early biosynthetic transition within gallate-related metabolism
	GaQA (II)	1.000	1.000	1.000	Medium-high (0.041)	Derivation of quinic acid flux toward secondary phenolic metabolites
	ChA	0.997	0.999	1.000	Medium-high (0.033)	Redirection toward hydroxycinnamic acids biosynthesis
*Flavonoids*	KRu	0.988	0.996	1.000	Medium-high (0.033)	SA-induced discriminating flavonol conjugated
	QRh	0.988	0.996	0.997	High (0.061)	Plasticity of the quercetin nucleus under SA
*Hydrolyzable tannins*	EAG (II)	0.999	1.000	1.000	Very high (0.136)	Structural biomarker of control and ellagic axis
	EAR (II)	0.996	1.000	0.999	Very high (0.124)	Basal state of ellagic metabolism
	MPEA	0.997	1.000	0.999	High (0.102)	Fine modulation of terminal ellagic derivatives induced by SA
	DGG (II)	0.993	0.995	0.999	Medium (0.028)	Rescheduling of the galloylated pattern within tannin pools
***Jasmonic acid***						
*Phenolic acids*	DCQA	0.992	0.972	0.997	Medium-high (0.045)	Advanced caffeoylquinic ester; reflects JA-dependent fine modulation of phenylpropanoid flux
	SQA	0.997	0.999	0.999	Medium-high (0.033)	Shikimate/quinic node modulating precursor availability
	ChA	0.994	0.998	0.997	Medium (0.029)	Caffeoylquinic intermediate linking QA and hydroxycinnamic acids
	QA	0.993	0.989	0.998	Medium (0.024)	Central quinic scaffold; reflects background restructuring of acceptor pools
	CaH	0.995	0.996	1.000	Medium (0.020)	Free hydroxycinnamic acid; contextual indicator of phenylpropanoid activation
*Flavonoids*	QG	0.982	0.998	0.994	Very high (0.110)	Core quercetin conjugate; indicates strong JA-induced redirection toward flavonol glycosylation
	C	0.982	0.994	0.994	High (0.098)	Structural bridge between flavonoids and tannin polymerization
	KRu	0.999	1.000	1.000	High (0.055)	Highly JA-sensitive flavonol rutinoside associated with defense-related responses
	QβG (I)	0.992	0.997	0.993	Medium-high (0.039)	Quercetin 3-O-β-glucuronide; reflects conjugation-based stabilization of flavonols
*Hydrolyzable tannins*	MPEA	0.998	1.000	0.999	Maximum (0.138)	Terminal ellagic derivative; key metabolic indicator of JA-driven redirection toward advanced ellagic metabolism
	EAR (II)	0.996	1.000	0.999	Very high (0.126)	Advanced ellagic conjugate; reflects robust JA modulation of late-stage ellagic forms
	HHDPdGG (II)	0.994	0.998	0.997	Medium-high (0.044)	Highly condensed ellagitannin (HHDP-digalloyl-glucose), reflecting advanced galloylation and oxidative coupling under JA treatment
	MGG (II)	0.976	0.996	0.996	Medium-high (0.034)	Galloylation intermediate; sensitive to JA-driven remodeling of gallotannin pools
***Abscisic acid***						
*Phenolic acids*	ChA	0.999	1.000	0.999	Medium (0.025)	Bridge metabolite linking quinic acceptors to caffeoyl conjugation; CQA rebalancing under ABA
	SQA	0.998	1.000	1.000	Very high (0.121)	Marker of phenylpropanoid rerouting toward sinapoylquinic conjugation; structural/defensive phenolic remodeling
	DCQA	0.993	0.994	0.998	Medium (0.020)	Advanced caffeoyl esterification state; indicate time × dose windows favoring di-ester accumulation
	GaHA (I)	0.986	0.995	0.993	Medium-high (0.032)	Early/structural shift in gallate-related pool; gatekeeper of galloyl chemistry under ABA
*Flavonoids*	KRu	0.992	0.998	0.997	High (0.051)	Discriminating flavonol conjugate; robust ABA control of rutinosylation (storage/defense phenotype)
	QG	0.989	0.998	0.994	High (0.074)	Reorganization of the quercetin-conjugate pool; ABA-driven channeling to glycosylated flavonols
	PB1	0.572	0.987	0.968	Maximum (0.198)	Primary discriminator of the proanthocyanidin axis; marker of flavan-3-ol dimerization/condensation under ABA
	ECG	0.983	0.996	0.993	Medium (0.023)	Galloylated flavanol; indicator of enhanced antioxidant structuring
*Hydrolyzable tannins*	EAR (II)	0.995	1.000	0.998	Very high	Advanced ellagic conjugate; marker of sustained ABA control over late ellagitannin maturation
	MPEA	0.998	1.000	0.999	Very high (0.114)	Terminal mixed ellagic derivative; sensitive integrator of ABA-driven remodeling of the HHDP-ellagic axis
	EAR (I)	0.980	0.989	0.990	Medium (0.025)	Complementary ellagic-state indicator; supports ellagic baseline tuning across time × dose

* based on MDA (Mean Decrease Accuracy).

## Data Availability

The datasets generated and analyzed during the current study are not publicly available but are available from the corresponding author upon reasonable request.

## Abbreviations

The following abbreviations are used in this manuscript:

ABA	Abscisic acid
CaH	Hydroxycinnamic acid
ChA	Chlorogenic acid
CQA	Caffeoylquinic acid
DCQA	Dicaffeoylquinic acid
DGG	Digalloylglucose
EAR	Ellagic acid rhamnoside
EAG	Ellagic acid glucose
ECG	Epicatechin gallate
GaHA	Galloyl hexoside acid
GaQA	Galloylquinic acid
HCT	Hydroxycinnamoyl-CoA: shikimate hydroxycinnamoyl transferase
HQT	Hydroxycinnamoyl-CoA: quinate hydroxycinnamoyl transferase
HHDP	Hexahydroxydiphenoyl
HHDPdGG	Hexahydroxydiphenoyl-digalloylglucose
JA	Jasmonic acid
KRu	Kaempferol rutinoside
MANOVA	Multivariate analysis of variance
MDA	Mean decrease accuracy
MGG	Monogalloylglucose
MPEA	Methylated pentoside ellagic acid
PB1	Procyanidin B1
PLS-DA	Partial least squares–discriminant analysis
QA	Quinic acid
QG	Quercetin glucoside
QβG	Quercetin β-glucuronide
RF	Random Forest
SA	Salicylic acid
SQA	Sinapoylquinic acid
